# Review of Layered Double Hydroxide (LDH) Nanosheets in Corrosion Mitigation: Recent Developments, Challenges, and Prospects

**DOI:** 10.3390/ma18061190

**Published:** 2025-03-07

**Authors:** Jintao Cao, Yangmin Wu, Wenjie Zhao

**Affiliations:** 1 State Key Laboratory of Advanced Marine Materials, Ningbo Institute of Materials Technology and Engineering, Chinese Academy of Sciences, Ningbo 315201, China; caojintao@nimte.ac.cn; 2 University of Chinese Academy of Sciences, Beijing 100049, China

**Keywords:** LDHs, corrosion protection, physical barrier, self-healing, chloride trapping effect, composite coating

## Abstract

Layered double hydroxides (LDHs) are a typical class of two-dimensional nanomaterials that present numerous possibilities in both scientific and practical applications. LDHs, with a layered structure and unique interlayer ion-exchange properties, can be utilized to prepare various functional coatings, showing great potential in the field of marine corrosion protection. In this review, the preparation approaches and properties of LDHs are first briefly introduced. Subsequently, various protection types based on LDH-based composite coatings for marine corrosion protection are highlighted, including physical barriers, self-healing, chloride trapping effects, and hydrophobic effects, respectively. Furthermore, critical factors influencing the anti-corrosion performance of composite coatings are discussed in detail. Finally, remaining challenges and future prospects for LDH-modified composite coatings in corrosion protection are proposed. This review provides a distinctive perspective on fabricating LDH-enhanced corrosion-resistant materials, contributing toward the development of multifunctional, intelligent anti-corrosion coatings for diverse applications.

## 1. Introduction

In recent years, as the scope and depth of ocean exploration have continued to expand, the importance of corrosion protection for engineering equipment employed in marine environments has become increasingly evident [1]. The ocean is a complex and demanding environment with multi-factorial coupling [2,3], including high salt levels, high temperature, high humidity, high hydrostatic pressure, dissolved oxygen, and microorganisms, which pose significant threats to metal-based structures and equipment. Therefore, it is imperative to apply appropriate strategies to alleviate the destruction triggered by corrosion. To mitigate the detrimental effects of corrosion, a number of methods, predominately including sacrificial anodes [4,5], corrosion inhibitors [6], and coatings [7,8], have been developed and employed for providing corrosion resistance. Among the strategies mentioned above, protective coatings, especially organic coatings, provide ease of application, low cost, and strong versatility, leading them to be regarded as one of the most promising methods. Organic coatings can act as a physical barrier on metal substrates, effectively preventing the infiltration of corrosion agents [9]. Unfortunately, organic coatings present numerous inherent micro-holes, owing to their rapid formation process, which accelerates coating failure and diminishes their ability to protect metals.

A significant number of studies have explored ways to improve the anti-corrosion properties of organic coatings. Among these strategies, incorporating fillers with a flake-like structure into the coating matrix is a valuable option. Typical flake-like fillers include mica flakes, glass flakes, and others, which are distributed and arranged to create a labyrinth effect, thus greatly enhancing the physical shielding performance of organic coatings. However, most of these fillers are micrometer-level, and in practical applications, a significant amount must be added to meet the required standards for corrosion protection. As a result, this not only increases the difficulty of dispersing fillers within the resin, but also damages the physical and chemical properties of the coating itself, thereby compromising its anti-corrosion effectiveness.

Nowadays, with the rapid development of micro–nano technology, two-dimensional (2D) nanomaterials have come to play an increasingly important role as fillers in anti-corrosive coatings, such as graphene and its derivatives, hexagonal boron nitride, and transition metal dichalcogenides. Firstly, these 2D layered nanomaterials can effectively fill defects within the coating, thereby improving its density. Furthermore, these nanosheets form a dense physical barrier layer in the coating matrix. Thirdly, the incorporation of nanofillers can improve the mechanical properties of the coating, thereby enhancing its anti-corrosion performance. Among these 2D nanomaterials, layered double hydroxides (LDHs) present a superior labyrinth effect, together with excellent ion-exchange performance, making them one of the most widely used nanofillers for achieving long-term corrosion protection. This ion-exchange capability can endow the coating with an excellent chloride trapping effect. In addition, the unique layered structure of LDHs provides them with an excellent ion capacity, and allows them to serve as a nanocontainer for storing corrosion inhibitors, capturing chloride ions while releasing corrosion-inhibiting molecules, thereby achieving integration of the coating’s physical barrier and self-healing properties. In addition to binary LDHs, ternary LDHs exist, which consist of three kinds of metal cations. In recent years, ternary LDHs such as ZnMgAl have also been applied in the field of corrosion protection [10]. Wang et al. reported that ZnMgAl LDH films presented excellent corrosion inhibition for aluminum foils [10]. Compared to binary LDHs, ternary LDHs allow for the precise control of properties by adjusting the types and proportions of metals. However, their synthesis conditions are more demanding, and the associated costs are higher. So far, LDHs have played an increasingly important role in corrosion protection coatings, and several reviews have been published on the anti-corrosive properties of LDH-reinforced organic coatings, with most focusing mainly on their preparation strategies and ion-exchange and corrosion-resistant properties [11,12,13]. Recently, Cao et al. published a systematic review which found that LDHs could be used in powders and films for corrosion protection [11]. Tabish et al. highlighted the current status of LDHs as nanocontainers for various inhibitors, offering long-term corrosion protection [12]. Bouali et al. elaborated that LDHs served as functional conversion layers and nanofillers to provide corrosion protection for aluminum alloys [13]. However, the ion-exchange properties of LDHs, and the crucial factors that determine their corrosion protection capabilities, have rarely been systematically discussed to help provide an in-depth understanding of the mechanisms of LDHs in corrosion protection. This review offers a comprehensive overview of recent advancements in LDH-reinforced organic coatings for corrosion protection. Firstly, the structure and preparation methods of LDHs are briefly introduced. Subsequently, the primary types of corrosion protection provided by LDH-reinforced organic coatings, such as physical barriers, self-healing, chloride trapping effects, and hydrophobic effects, are emphasized. In addition, the key factors that determine their corrosion-resistant performance are discussed in detail, resulting in an in-depth understanding of the corrosion mechanisms of composite coatings. Finally, remaining challenges and future prospects for LDH-modified composite coatings in corrosion protection are proposed. This review provides an innovative viewpoint on designing robust, sustainable, and low-cost LDH-modified anti-corrosive composite coatings, which potentially offer options or inspiration in the fields of industrial corrosion, marine corrosion, and beyond.

## 2. Physicochemical Characteristics

### 2.1. Structure and Properties

**Figure 1:**
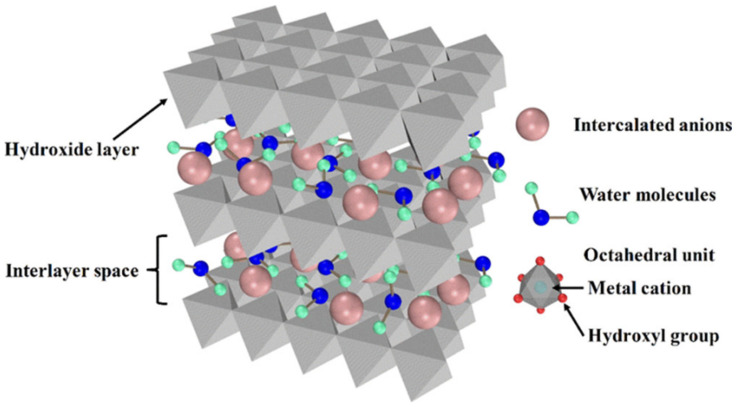
Schematic structure of LDHs. Adapted with permission from Reference [14]. Copyright 2018, American Chemical Society.

LDHs are generally described as a class of anionic clay minerals with the general formula [M^2+^_1−x_M^3+^_x_(OH)_2_]^X+^(A^n−^_x/n_)·mH_2_O, and their structure is shown in Figure 1, where M^2+^ and M^3+^ represent divalent and trivalent metal cations, and A^n−^ represents interlayer anions [14]. Among them, M^2+^ ions typically include Mg^2+^, Zn^2+^, and Cu^2+^, while M^3+^ ions include Al^3+^, Fe^3+^, and Mn^3+^. The cation layer consists of an octahedral structure formed by divalent and trivalent metal ions, which are interconnected by hydroxyl groups (OH^−^) to form a planar layer. Since the radius of the trivalent metal ion is smaller than that of the divalent metal ion, this substitution results in an uneven distribution of positive charge within the positively charged layer, resulting in a net positive charge throughout the layer. Interlayer anions and water molecules are located between the positively charged layers, where they interact with the metal hydroxide layer through electrostatic attraction to achieve charge balance. The bonding strength of electrostatic interactions is significantly weaker than that of chemical bonds. These phenomena enhance the ion-exchange behaviors of LDHs, allowing them to achieve intelligent coating protection, including chloride ion trapping and inhibitor release.

In general, LDHs present a strong affinity with carbonate ions (CO_3_^2−^), making them difficult to remove. At present, there are only three methods to eliminate them. Firstly, CO_3_^2−^ can be removed from LDHs in a NaNO_3_ methanol solvent. Secondly, CO_3_^2−^ can be eliminated in an acid solution via the production of carbon dioxide. Thirdly, CO_3_^2−^ can be removed in a concrete environment by forming CaCO_3_ [15,16].

### 2.2. Preparation

**Figure 2:**
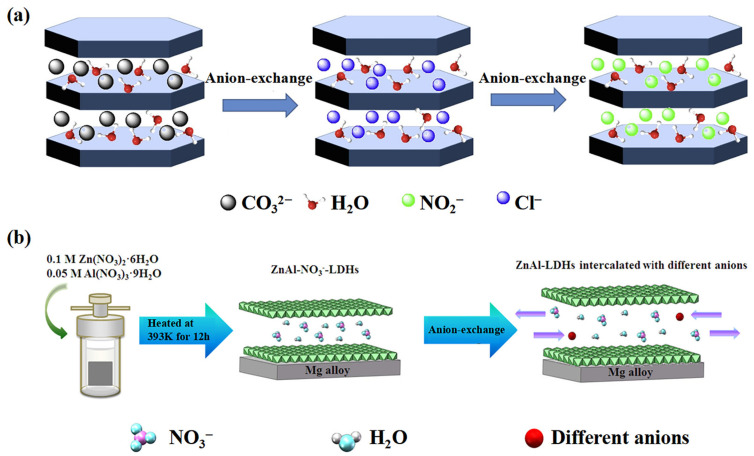
(**a**) The anion exchange process for the preparation of nitrite ion-intercalated MgAl-LDHs. Adapted with permission from Reference [29]. Copyright 2020, Elsevier. (**b**) A diagram of the experimental process for the formation of ZnAl-LDH films intercalated with different anions. Adapted with permission from Reference [31]. Copyright 2019, Elsevier.

Conventional methods for the preparation of LDHs mainly include hydrothermal synthesis [17,18,19], co-precipitation [20,21,22], in situ growth [23,24,25], and burned reduction [26]. The methods mentioned above have been elaborated upon extensively in the literature and in various reviews; this review specifically focuses on the ion-exchange method. The ion-exchange strategy is mainly achieved through the structural modulation of LDHs. Specifically, this process is accomplished by immersing the precursor in a solution containing the target anion, which promotes the exchange of the original anion and the new anion between the layers, thereby obtaining a specific LDH. This process is usually carried out at specific temperatures and pH to promote exchange efficiency [27,28]. The ion-exchange method is simple, gentle, and suitable for introducing various kinds of functional anions, thus conferring new properties to LDHs. As shown in Figure 2a, Su et al. removed CO_3_^2−^ from an LDH precursor by acidic oscillations to obtain LDH-Cl^−^, which was then dissolved in a nitrous acid solution. Finally, nitrite ion-intercalated MgAl LDHs were obtained via the ion-exchange method [29]. Furthermore, the dispersibility of LDHs is crucial for the preparation of high-performance protective coatings. In addition to traditional surface modification methods, Yu et al. used a hydrostatic pressure-assisted ultrasonication method to trigger enhanced shock waves, achieving ultrasonic bubble implosion, and thereby obtaining uniform dispersion of carbon nanotubes [30]. This strategy is also effective for dispersing a variety of nanofillers, such as LDHs.

In addition, LDHs intercalated with different anions can be directly obtained by intercalating ions. As shown in Figure 2b, different types of ZnAl LDHs can be synthesized by intercalating various anions, such as Cl^−^, VO_4_^3−^, PO_4_^3−^, and MoO_4_^2−^ [31]. In summary, inhibitor molecules can be intercalated into the interior of LDHs through an ion-exchange strategy, exhibiting excellent corrosion protection capabilities.

## 3. Anti-Corrosion Mechanisms

LDHs exhibit excellent ion-exchange capacity, making them widely utilized in the field of corrosion protection [32,33,34,35,36]. The interlayer anions of LDHs can be connected to laminates through electrostatic interactions, and can react with external ions under external stimulation. Through ion-exchange, corrosion inhibitors can be incorporated into LDHs to provide self-healing protection, which is the primary protection mechanism of LDHs [27,28,37,38,39,40]. Furthermore, LDHs show excellent chloride trapping effects, which is another main mechanism for corrosion protection. In addition, LDHs present layered structures, which effectively prevent the penetration and diffusion of corrosion-related species into coatings, thereby raising their anti-corrosion performance. Spherical and rod-shaped fillers are often used to enhance the mechanical properties of organic coatings, due to their high specific surface area and mechanical strength. In the field of corrosion protection, physical barriers are a crucial factor. Two-dimensional nanosheets can effectively delay or even prevent the penetration and diffusion of corrosion-related species, owing to their layered structure [14]. In this manner, the protected metals can maintain their original states for an extended period, thereby prolonging their service life. In addition, nanosheets can improve the mechanical properties of composite coatings to a certain extent [41]. Therefore, as the application environment becomes increasingly demanding, 2D nanosheet-reinforced protective coatings play an increasingly important role. Also, some LDHs exhibit a nest-like structure, which can endow the organic coating with hydrophobic properties, preventing the adhesion and penetration of corrosion-related molecules, and thus providing superior protection performance [11,42,43]. Owning to the fact that the corrosion behaviors and protection mechanisms of LDH-reinforced organic coatings are quite different, their related mechanisms are discussed separately in this section.

### 3.1. Physical Barrier

LDHs, as one of the most representative 2D nanomaterials, possess a high aspect ratio and high impermeability, which can endow organic coatings with outstanding physical barrier properties. In addition, as nano-scale material, LDHs can effectively fill micro-defects caused by the rapid curing of organic coatings, thereby improving their density.

Bai et al. incorporated an LDH into an epoxy coating to enhance the coating’s physical barrier characteristics, and found that the low-frequency impedance of the LDH/epoxy coating improved by about two orders after 40 days of immersion in saline water (3.5%) [44]. However, a single LDH is insufficient for enhancing the corrosion protection performance of organic coatings, and fails to meet the service requirements of harsh environments.

**Figure 3:**
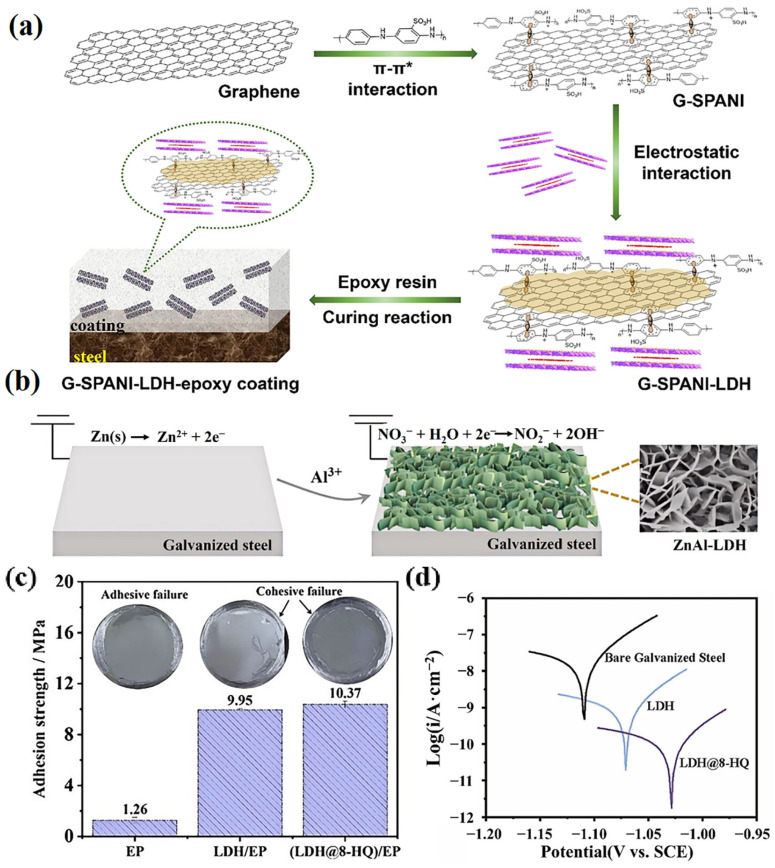
(**a**) Schematic of preparation of graphene–LDH nanohybrid via sulfonated polyaniline mediated self-assembly approach, and G-SPANI-LDH epoxy coating. Adapted with permission from Reference [45]. Copyright 2021, Elsevier. (**b**) Schematic illustration of in situ deposition of ZnAl-LDH coatings on galvanized steel by two-step electrochemical method, (**c**) pull-off test results of different samples, and (**d**) potentiodynamic polarization curves of different samples after immersion in 3.5 wt% NaCl aqueous solution for 30 min. Adapted with permission from Reference [47]. Copyright 2024, Elsevier.

Inserting functional factors (such as corrosion inhibitors) into LDHs, or preparing heterojunction structures, are effective strategies for enhancing the corrosion protection performance of composite coatings. Su et al. developed a sulfonated polyaniline crosslinked graphene–LDH nanohybrid (G-SPANI-LDH) to improve the corrosion protection performance of an epoxy coating (Figure 3a) [45]. Electrochemical parameters indicated that the low-frequency impedance of the composite coating remained around 10^8^ Ω·cm^2^ after 70 d immersion in 3.5 wt% NaCl solution, which was one order of magnitude higher than that of the epoxy coating, suggesting remarkable corrosion protection performance. This phenomenon could be due to the improved physical barrier properties of G-SPANI-LDH. Cai et al. prepared a Ti_3_C_2_T_x_ MXene-functionalized MgAl LDH (Ti_3_C_2_T_x_@MgAl-LDH) heterostructure, and the corrosion resistance performance of the Ti_3_C_2_T_x_@MgAl-LDH/epoxy coating realized a significant improvement, based on the physical barrier effects of MgAl LDH and Ti_3_C_2_T_x_ nanosheets [46]. After immersion in 3.5 wt% NaCl for 21 days, the low-frequency impedance of the Ti_3_C_2_T_x_@MgAl-LDH-reinforced epoxy coating remained at 3.9 × 10^6^ Ω·cm^2^, which was almost two times greater than that of the epoxy coating (2.0 × 10^6^ Ω·cm^2^).

Due to their lamellar structures, LDHs can endow organic coatings with exceptional physical barrier capabilities, which is the fundamental function of LDHs in enhancing corrosion protection performance. In addition, the amount, alignment, and orientation of LDHs in the coating matrix directly determine the anti-corrosion performance of composite coatings.

Xu et al. employed an in situ, two-step electrochemical strategy to obtain an LDH with a vertically grown morphology, and then incorporated it into an epoxy coating to improve its corrosion resistance (Figure 3b) [47]. Owing to its unique morphology, the LDH was able to yield a significantly “concave filled structure” of the epoxy matrix, which largely improved the adhesion strength between the underlying metal substrate and the epoxy coating, thus boosting its anti-corrosive properties (Figure 3c,d). In addition, they indicated that this vertical structure not only avoided the self-agglomeration of the LDH, but also resulted in greater contact with the polymeric matrix; thus, there were more hydrogen bonds between the LDH nanosheets and the resin backbone, which could enhance the anti-corrosion capability [48].

Physical barrier performance is the fundamental function of LDH-reinforced organic coatings. To achieve more effective corrosion protection, it is essential to develop multifunctional coatings based on physical shielding properties.

### 3.2. Chloride Trapping Effect

Chloride ions are a critical factor in accelerating the corrosion of metals, especially in marine and chlorinated environments. They compromise the protective layer on the metal surface, resulting in pitting and stress corrosion cracking. This phenomenon is due to the fact that Cl^−^ is involved in electrochemical reactions that lower the corrosion potential of the metal, thereby exacerbating metal corrosion. The mechanism of the chloride trapping effect mainly relies on the special layered structure of LDHs. When Cl^−^ enters the LDH, it can exchange with internal intercalated ions, thereby reducing the Cl^−^ concentration and prolonging the service life of the composite coatings.

**Figure 4:**
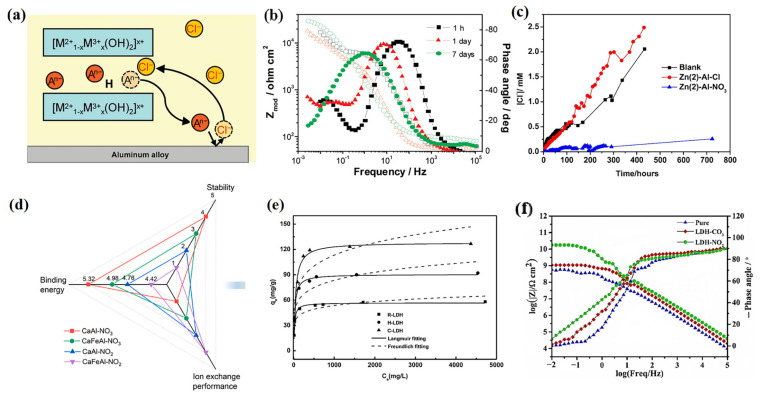
(**a**) Schematic diagram of chloride trapping effect. (**b**) Bode plots of impedance measured on bare AA2024 in ZnAl−NO_3_^−^+ 0.05 M NaCl. Adapted with permission from Reference [49]. Copyright 2009, American Chemical Society. (**c**) Cl^−^ permeability through coating as function of time. Adapted with permission from Reference [50]. Copyright 2012, Elsevier. (**d**) Stability and ion-exchange performance of LDHs in different testing solutions. Adapted with permission from Reference [38]. Copyright 2024, Elsevier. (**e**) Equilibrium isotherms of chloride adsorption on R-LDH, C-LDH, and H-LDH. Adapted with permission from Reference [52]. Copyright 2019, Elsevier. (**f**) Bode diagrams of LDH-reinforced epoxy coating soaked for 5 days. Adapted with permission from Reference [29]. Copyright 2020, Elsevier.

Poznyak et al. synthesized Zn-Al and Mg-Al LDHs containing quinaldate and 2-mercaptobenzothiazolate anions through anion-exchange reactions [49]. Spectrophotometric measurements suggested that the LDHs could effectively trap chloride ions, thus releasing inhibitor molecules within the body and improving the corrosion protection performance of an AA2024 aluminum alloy (Figure 4a). Additionally, a Bode plot indicated that the low-frequency impedance of ZnAl-NO_3_^−^ remained relatively stable over a week, demonstrating significantly improved corrosion protection (Figure 4b).

Obviously, LDHs can release an equivalent amount of intercalated molecules, primarily consisting of corrosion inhibitors, when trapping chloride ions. Based on the chloride trapping effect of LDHs, the concentration of corrosive agents can be significantly reduced, thereby extending the service life of the metal substrate. Tedim et al. proposed that an Zn-Al LDH intercalated with nitrate ions could effectively improve the corrosion protection ability of a polymeric coating, by drastically decreasing the permeability of Cl^−^ into the coating matrix (Figure 4c) [50]. In addition, they found that a coating containing LDH-NO_3_ presented lower chloride ion permeability (the permeability decreased by around 20 times), which was mainly attributed to the ion-exchange reaction of the LDH. Specifically, it effectively trapped chloride ions while releasing NO_3_ corrosion inhibitors in a NaCl solution.

In fact, the process of chloride trapping through ion-exchange usually requires external stimulation. Some anions exhibit a high affinity for the layered structure, greatly reducing the chloride trapping efficiency, which also determines the corrosion protection ability of organic coatings. According to Miyata [51], nitrate ions (NO_3_^−^) exhibit highly reactive characteristics and readily exchange with chloride ions, resulting in an organic coating that demonstrates excellent chloride trapping capabilities.

Xu et al. evaluated the chloride trapping and corrosion inhibition effects of NO_3_^−^- and NO_2_^−^-intercalated MgAl LDHs in saturated calcium hydroxide solution by electrochemical impedance spectroscopy (EIS) measurement [22]. It was found that the chloride uptake load and chloride concentration at equilibrium exhibited a significant non-linear relationship, and the maximum chloride uptake of NO_3_^−^-intercalated LDHs was 3.014 mmol/g, while the maximum value for NO_2_^−^-intercalated LDHs was only 2.510 mmol/g. Owing to the chloride trapping effect, the prepared LDHs presented a superior corrosion protection ability. In addition, Xu et al. indicated that the ion-exchange process of LDHs was closely related to the interlayer spacing and binding energy between the intercalated cations [38]. They gained insights into the relationship between binding energy, stability, and ion-exchange behavior through binding energy simulations and density functional theory (DFT) calculations (Figure 4d). The chloride trapping rate of LDH-NO_3_^−^ with a higher binding energy was greater than that of LDH-NO_2_^−^ with a lower binding energy, and the release rate of the intercalated ions was higher due to the lower binding energy of NO_2_^−^ to the cationic layer.

Different preparation methods also have varying effects on the chloride trapping effects of LDHs. Zuo et al. investigated the chloride trapping ability of NO_2_^−^-intercalated LDHs prepared using three different methods, including calcination–rehydration, hydrothermal, and co-precipitation methods [52]. The LDHs prepared by the co-precipitation method exhibited the highest chloride trapping capacity (Figure 4e), thus releasing a greater amount of corrosion inhibitors. Su et al. prepared a nitrite ion-intercalated MgAl LDH (LDH-NO_2_), and the anti-corrosion performance of the LDH-NO_2_/epoxy coating was greatly enhanced, based on the chloride trapping effect of the LDH and the corrosion inhibition of the NO_2_ [29]. After 5 days of immersion in 3.5 wt% NaCl solution, the low-frequency impedance of the LDH-NO_2_/epoxy coating was 1.315 × 10^10^ Ω/cm^2^, which was two orders of magnitudes higher than that of a pristine LDH/epoxy coating (Figure 4f). When the coating was cracked, LDH-NO_2_ could effectively exert a chloride trapping effect and release NO_2_ molecules, providing the composite coating with excellent corrosion protection performance.

In general, relying solely on the chloride trapping effect makes it challenging to effectively meet the actual demands for corrosion protection [53]. When designing high-performance corrosion protection coatings, it is essential to combine the chloride trapping effect with other modes of protection in order to ensure long-term corrosion resistance.

### 3.3. Self-Healing

As typical nanocontainers, LDHs are capable of loading a significant quantity of corrosion inhibitor molecules. When a coating is damaged or scratched, corrosive ions can penetrate the interior of the coating through the defect, triggering the release of corrosion inhibitors stored within the nanocontainers. Subsequently, the corrosion inhibitors can react with the metal substrate to form a stable protective layer, slowing down the destruction of corrosion-related species, achieving self-healing properties.

The corrosion inhibitors (anions) mainly enter the LDH in the form of intercalation, and then are released in response to external stimulation. These anions can be divided into two broad categories, inorganic and organic anions, based on their chemical properties and corrosion inhibition behavior. This classification helps to optimize the corrosion protection of LDHs by allowing for the selection of an appropriate anion based on the application requirements and environmental conditions. Inorganic anions are usually more stable and have a long-lasting corrosion inhibition effect, while organic anions may provide a faster corrosion inhibition response. Inorganic anions inhibit corrosion by forming a stable protective film, while organic anions may interact with metal surfaces through chemisorption or bonding. In addition, inorganic and organic anions have different sensitivity to environmental change, which affects their release rate and corrosion inhibition efficiency (self-healing properties). In general, inorganic anions mainly include vanadate [54,55], molybdate [56,57], tungstate [58], and phosphate [59]. Organic anions mainly include mercaptobenzothiazole (MBT) [60,61], benzoic acid [35,62], 8-hydroxyquinoline (8-HQ) [63,64], and aspartic acid [65,66,67]. We elaborate on these two types of corrosion inhibitors below.

#### 3.3.1. Inorganic Anions

A ZnAl-LDH-containing decavanadate intercalation was synthesized by Buchheit et al. to protect an aluminum alloy substrate [68]. Salt spray test and X-ray diffraction (XRD) techniques confirmed that the release of decavanadate from the ZnAl-LDH could effectively protect the underlying AA2024 aluminum alloy substrate. At the same time, it could effectively exert a chloride trapping effect while releasing inhibitors, and further improve the corrosion protection ability. The low-frequency impedance value of the as-prepared coating containing decavanadate-intercalated LDHs remained above 10^6^ Ω·cm^2^ after 100 h immersion in 0.5 M NaCl solution. However, for the epoxy coating without LDHs, the low-frequency impedance value dropped immensely, from 10^6^ (20 h) to 10^4^ Ω·cm^2^ after 100 h immersion.

**Figure 5:**
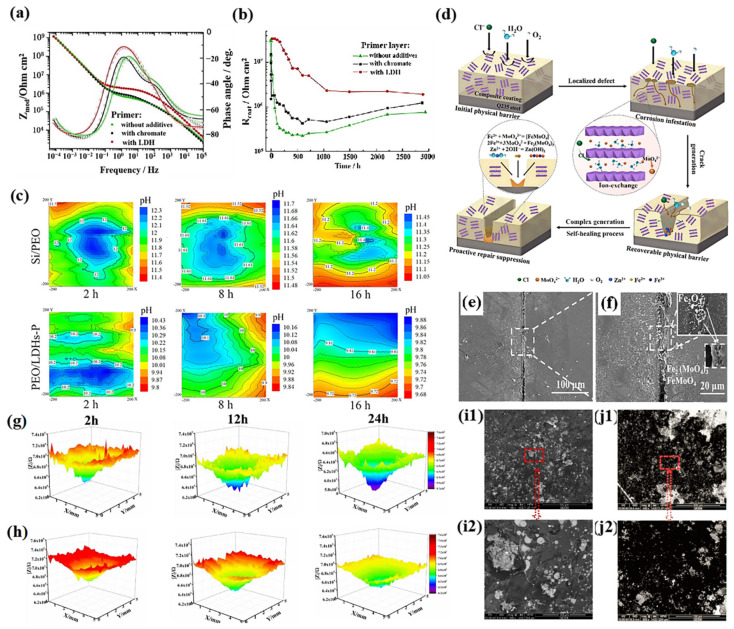
(**a**) Bode plots of AA2024 with and without LDH coatings. (**b**) Time evolution of pore resistance of top coat and primer layer without additives and with chromate and LDH additives, which were deposited on AA2024 samples, during immersion in 0.5 M NaCl. Adapted with permission from Reference [69]. Copyright 2010, Elsevier. (**c**) pH distributions around defect after different times of immersion in 0.05 M NaCl. Adapted with permission from Reference [72]. Copyright 2022, Elsevier. (**d**) Schematic illustration of triple protection mechanism of EP/LDH-MoO_4_ in sodium chloride solution. (**e**,**f**) SEM images of scratches on EP/LDH-MoO_4_. Adapted with permission from Reference [73]. Copyright 2023, American Chemical Society. LEIS test for (**g**) pure epoxy coatings and (**h**) LDH-NO_2_^−^ epoxy coatings, and corrosion morphology of steel under (**i1**,**i2**) pure coating and (**j1**,**j2**) LDH-NO_2_ coating. Adapted with permission from Reference [29]. Copyright 2020, Elsevier.

Zheludkevich et al. used metavanadate intercalation to synthesize LDH-based nanocontainers [69]. The impedance value at low frequencies of LDHs containing metavanadate was slightly higher than that of chromium chloride in 0.5 M NaCl (Figure 5a). The stability of the protective layer, as well as the long-lasting corrosion protection, was improved by the incorporation of metavanadate (the resistance of the LDHs with vanadate intercalation was 2.4 × 10^9^ Ω cm^2^, which was slightly higher than that of chromate intercalation at 1.9 × 10^9^ Ω cm^2^ (Figure 5b)). To improve the loading capacity of the inhibitor, Yasakau et al. used a bilayer system, combining pretreated LDH with a hybrid sol–gel coating, to achieve the long-term release of corrosion inhibitor ions [70]. The vanadate release rate was effectively reduced (about 15 times lower compared to pure LDH), thus prolonging the corrosion inhibition time.

The potential of phosphate-loaded LDHs for corrosion protection has also been demonstrated in several studies [71,72]. The main corrosion inhibition mechanisms are derived from the release of PO_4_^−^ inhibitors and the trapping behavior of chloride ions. Zhang et al. investigated the local self-healing behavior of LDH composite coatings with phosphate inhibitor intercalation using the scanning ion-selective electrode technique [72]. Silicate-based plasma electrolytic oxidation (PEO) specimens were selected as a reference. As shown in Figure 5c, the pH values of the silicate-based PEO and phosphate-based PEO specimens decreased continuously with an increase in immersion time, but the pH value of the central region of the Si/PEO (12.3) specimen was much larger than that of the PEO/LDHs-P (10.4). This observation strongly indicated that the PEO/LDHs-P presented a superior corrosion protection ability, owing to its inhibition effect. With the increase in immersion time, the micro-defects on the PEO/LDHs-P composite coating were completely healed after 16 h, and the defects on the Si/PEO coating could still be observed clearly.

In comparison to vanadate and phosphate, molybdate-intercalated LDHs show more prominent corrosion protection enhancement effects on organic matrices. MoO_4_^2−^ presents a superior ion-exchange capacity compared to other intercalated ions. Li et al. verified that the corrosion protection of LDH-MoO_4_^2−^ was more effective than that of ZnAl-LDH-NO_3_^−^ and ZnAl-LDH-PO_4_^−^ [73]. This result was attributed to the larger radius of molybdate ions, expanding the interlayer spacing of the LDHs and further promoting the release of inhibitor molecules (self-healing property). By generating insoluble FeMoO_4_ or Fe_2_(MoO_4_)_3_ upon coming into contact with the metal substrate (Figure 5d), MoO_4_^2−^ exhibited stronger self-healing properties (Figure 5e,f). Around the scratches, the micro-defects could be effectively healed, owing to existence of MoO_4_^2−^, thereby presenting superior self-healing performance.

Compared with other kinds of anions, inorganic anions are relatively small in size and have a greater charge density, making it easier to insert them into interlayer gaps, where they can exist more stably within LDHs. Inorganic anions such as NO_3_^−^ and PO_4_^3−^ usually provide superior self-healing protection, and are able to form a protective film on the metal surface, preventing the corrosive species from coming into direct contact with the metal, and thus slowing down or inhibiting the corrosion reaction. This protective film is usually dense and stable, and can provide effective corrosion protection over a long time. However, the inhibitor release efficiency of such types of LDHs may not be sufficient, which limits their actual application.

Localized electrochemical impedance spectroscopy (LEIS) is a powerful tool to characterize the self-healing performance of coatings [29]. As shown in Figure 5g,h, red and dark-blue colors indicate high impedance and low impedance, respectively. The impedance value at the defects monotonically decreased with increasing immersion time (Figure 5g), indicating poor corrosion resistance of the epoxy coating. Figure 5i shows the corrosion morphology of Q235 metal coated with an epoxy coating in which corrosion pits and micro-cracks are visible. For the LDH-NO_2_^−^ epoxy coating, the impedance value around the defects was higher than that at the initial stage (2 h) after 24 h of immersion in 3.5 wt% NaCl solution (Figure 5h), indicating that NO_2_^−^ oxidized the metal substrate and produced a high impedance value of the passive protective layer (Figure 5j), which endowed the coating with excellent self-healing properties.

#### 3.3.2. Organic Anions

**Figure 6:**
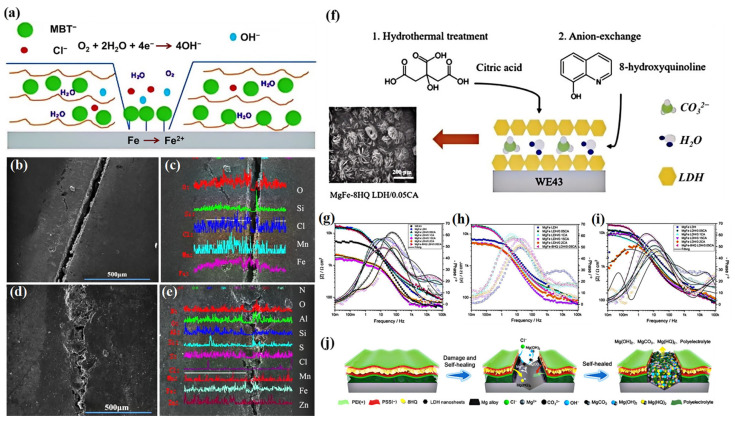
(**a**) Self-healing mechanism of EP-MBT/LDH coating. SEM and line scanning of scratched coatings after immersion for 13 h: (**b**,**c**) EP coating, (**d**,**e**) EP-MBT/LDH coating. Adapted with permission from Reference [76]. Copyright 2014, Springer Nature. (**f**) Schematic of corrosion inhibition effect of 8HQ/CA-LDH. (**g**–**i**) EIS plots of 1 h, 1-day, and 7-day immersion of different LDHs. Adapted with permission from Reference [64]. Copyright 2022, Elsevier. (**j**) Self-healing mechanism of PEI/PSS/8HQ/PSS/PEI coating on Mg alloy. Adapted with permission from Reference [77]. Copyright 2024, American Chemical Society.

In contrast to inorganic anions, most organic anions are macromolecular in structure, and their insertion increases the interlayer spacing of LDHs. Organic ions usually exhibit larger intermolecular forces and lower charge densities, which may result in their lower stability between the layers of LDHs, but they facilitate a rapid release rate. MBT is considered to be one of the most widely used organic corrosion inhibitors [49,74,75,76], and mainly forms a chelate complex film by interacting with the metal surface as shown in Figure 6a. EP coatings display no healing ability, and EP-MBT/LDH composite coatings present significant self-healing ability for micro-defects (Figure 6b–e).

Another organic corrosion inhibitor is 8HQ, which works by impeding both anodic and cathodic dissolution reactions. Tan et al. constructed a composite coating consisting of Mg-Fe LDH-intercalated 8HQ and a citric acid-modified layer on a Mg WE43 alloy using electrodeposition, hydrothermal, and anion-exchange techniques (Figure 6f) [64]. As shown in their EIS plots (Figure 6g–i), the MgFe LDH/0.05 citric acid (CA) and MgFe-8HQ LDH/0.05 CA samples maintained a high phase angle after immersion in Hank’s solution for 7 days, indicating superior corrosion protection ability. The CA endowed the MgFe LDH coating with remarkable physical barrier performance, while the 8HQ intercalation layer further enhanced the self-healing performance. Xie et al. also suggested that the LDH-8HP-reinforced coating presented excellent self-healing ability due to the release of 8HQ molecules (Figure 6j) [77].

When the interlayer ions of LDHs are organic anions, the organic ions usually have higher chemical activity, and can form stronger chemical bonding with the metal, thus providing a protective layer and effectively inhibiting direct contact and reactions between the corrosive species and the metal. In addition, compared with inorganic anions, organic anion-intercalated LDHs exhibit larger interlayer spacing, which can achieve an efficient ion release rate and corrosion inhibition response. There are various types of organic anions, and it is possible to obtain corrosion inhibitors with different properties by changing the functional groups and molecular structure, such as self-healing and hydrophobicity features. This versatility opens up more possibilities to meet specific application requirements.

Inorganic corrosion inhibitors provide greater stability compared to organic corrosion inhibitors. They can be used in a wider range of harsh environments, and are better suited for high/low temperatures, high hydrostatic pressure, and high salinity. Organic corrosion inhibitors, however, contain rich functional groups that can form chemical bonding with the metal substrate or create a physical adsorption layer, resulting in a stronger corrosion inhibition ability. There have been a number of studies on fabricating the hybrid intercalation of LDHs with inorganic and organic anions, which can produce a synergistic effect [75,78,79]. Inorganic anions provide stable corrosion inhibition, while organic anions improve the rapid response and environmental adaptability of the composite coating, resulting in more effective self-healing.

### 3.4. Hydrophobic Effect

Hydrophobic properties are an important feature that improves the corrosion protection of LDH-reinforced organic coatings, effectively inhibiting or even preventing the infiltration and diffusion of corrosion-related species so as to improve the coatings’ corrosion protection ability. LDHs with low surface energy can be constructed by using the characteristics of ion-exchange, which endows coatings with outstanding hydrophobic properties. Composite coatings with hydrophobicity properties are one of the most effective strategies for mitigating metal corrosion.

**Figure 7:**
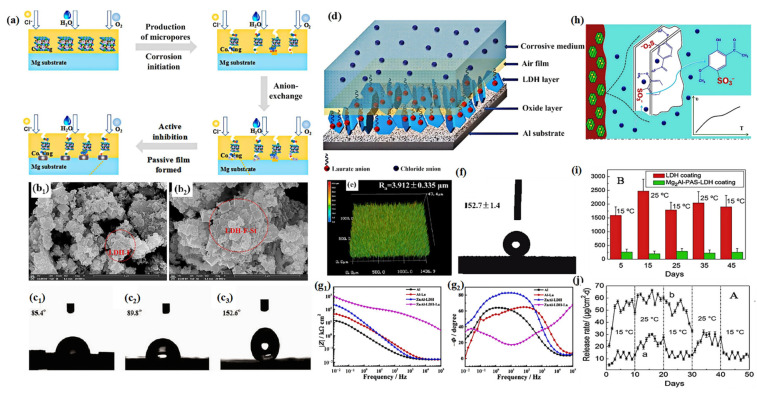
(**a**) Schematic representation of anti-corrosion mechanism for LDH-F-St-EP coatings, (**b_1_**,**b_2_**) SEM images of LDH-F and LDH-F-St, and (**c_1_**–**c_3_**) water CAs of pure EP, LDH-F-EP, and LDH-F-St-EP. Adapted with permission from Reference [80]. Copyright 2021, Elsevier. (**d**) Schematic illustration of corrosion protection mechanism of ZnAl-LDH-La, (**e**) laser microscopy images of ZnAl-LDH-La, (**f**) water CA of ZnAl-LDH-La, and (**g1**,**g2**) Nyquist plots of different samples in 3.5 wt% NaCl solution after immersion for 1 h. Adapted with permission from Reference [14]. Copyright 2018, American Chemical Society. (**h**) Anti-fouling principle of PAS-intercalated LDHs, (**i**) density of *Ulva* spores attached to LDH and Mg_2_Al-PAS-LDH coatings at 15 and 25 °C vs. their release days in 3.5% NaCl solution, and (**j**) release rate of Mg_2_Al-PAS-LDH and pristine PAS coatings in 3.5% NaCl solution at 15 and 25 °C. Adapted with permission from Reference [81]. Copyright 2017, Elsevier.

Cao et al. used stearate anion (St)-modified F^−^ intercalation MgAl-LDH nanosheets (LDH-F-St) to endow an epoxy coating with hydrophobic properties and superior anti-corrosion performance (Figure 7a) [80]. After modification with sodium stearate, the morphology and structure were not changed (Figure 7b), and the LDH-F-St-EP showed superhydrophobicity, with a contact angle (CA) as high as 152.6°, as shown in Figure 7c. Cao et al. prepared an LDH-La coating by hydrothermally intercalating laurate into the interlayers of LDHs to achieve a hydrophobic effect (Figure 7d) [14]. As shown in Figure 7e,f, owing to the modification of laurates (La), the composite coating presented a rougher surface with superhydrophobicity (~153°). The corrosion behavior of ZnAl-LDH-La was evaluated by EIS. As shown in Figure 7g, the low-frequency impedance modulus of the ZnAl-LDH-La composite coating was nearly two orders of magnitude higher than that of the Al substrate.

In addition, the excellent anti-fouling performance of the coating can also be enhanced by incorporating an intercalation agent. Yang et al. intercalated sodium paeonolsilate (PAS) into LDHs to achieve superior anti-fouling properties [81]. They characterized the anti-fouling performance of the PAS intercalation through a staining experiment of *Ulva* spores, and its anti-fouling principle is shown in Figure 7h. As shown in Figure 7i,j, for a pristine PAS coating, the attachment density of zoospores at around 25 °C was much greater than that at around 15 °C, indicating that the higher temperature caused more serious biofouling. The Mg_2_Al-PAS-LDH coating presented superior resistance to the settlement of *Ulva* spores, both at around 15 and 25 °C. It is becoming an increasingly important trend to develop multifunctional protective coatings by combining their hydrophobic properties with other characteristics.

The anti-corrosion performance of LDHs with different intercalating ions is summarized in Table 1. According to the table, it can be concluded that the corrosion protection performances of LDHs with various structural types are different. The anti-corrosion performances of LDHs depends on their structural types, such as whether they have interlayer anions or metal cations. In addition, the corrosion protection performance of LDHs is closely related to the immersion time and the service solution.

## 4. Main Factors Affecting Anti-Corrosion Properties of LDHs

To further advance the corrosion protection performance, it is essential to conduct a thoroughly investigation of the main factors influencing the corrosion protection behavior of LDHs. The corrosion protection capabilities of LDHs are mainly related to their unique structures, and are mainly affected by driving force and interlayer spacing. By analyzing these influencing factors, it is possible to optimize the synthesis of LDHs and design multifunctional anti-corrosion coatings, resulting in robust corrosion protection.

### 4.1. Driving Force

The corrosion protection behavior of LDHs is closely related to their ion-exchange characteristics. The driving force can affect the ion-exchange capacity to a large extent, so the driving force can indirectly determine the anti-corrosion ability of LDHs. The effect of the driving force on the ion-exchange capacity of LDHs interlayers has been investigated for cationic laminates and intercalated anions. Even for the same intercalation anion, the driving force between various metal cations and intercalation anions was different. Chen et al. synthesized a series of M^2+^ (Mg, Ca, Zn)-Al-NO_3_^−^ LDHs by a hydrothermal method [98]. Under the chloride binding capacity test, it was demonstrated that Zn^2+^, as the cation of LDHs, possessed the highest chloride binding and ion-exchange capacity compared to Ca^2+^ and Mg^2+^. Therefore, Zn-Al-NO_3_ LDHs enable more effective chloride trapping and release corrosion inhibitors in corrosion protection.

For the same type of ZnAl-LDH, Li et al. demonstrated that EP coatings containing ZnAl-LDH-MoO_4_^2−^ and ZnAl-LDH-PO_4_^3−^ had stronger corrosion protection and self-healing performance than those of EP coatings containing LDH-NO_3_^−^ [73]. This result was attributed to the trapping effect of corrosion anions between the LDH layers achieved by the inhibitor ions, inhibiting direct contact between aggressive species and the Q235 substrate. The driving force can affect the stability and ion-exchange ability of LDH interlayer anions, thus affecting the corrosion protection ability (mainly the chloride trapping ability).

**Figure 8:**
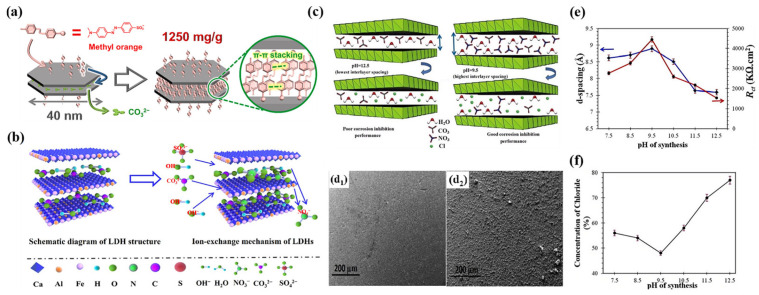
(**a**) Schematic representation of substitution of CO_3_^2−^ by MO. Adapted with permission from Reference [99]. Copyright 2021, Elsevier. (**b**) Layer spacing of LDHs with different arrangements of intercalated anions. Adapted with permission from Reference [100]. Copyright 2021, Elsevier. (**c**) Mechanistic description of corrosion inhibition performance of LDHs synthesized at pH = 9.5 ± 0.5 (highest interlayer spacing) and pH = 12.5 ± 0.5 (lowest), assigned to good and poor performance. SEM images of mild steel panels immersed in extract solutions after 24 h immersion: (**d_1_**) LDH synthesized at pH = 12.5, and (**d_2_**) LDH synthesized at pH = 9.5. (**e**) Variation in interlayer spacing (d-spacing) values and charge transfer resistance of Zn-Al-NO_3_-LDH at different pH values. (**f**) Concentration of chlorine in solution after 24 h immersion of mild steel. Adapted with permission from Reference [20]. Copyright 2020, Elsevier.

The driving force mainly consists of electrostatic interaction, hydrogen bonding, van der Waals forces, and ligand bonding. Electrostatic interactions play a key role in the ion-exchange behavior between cationic laminates and intercalated anions of LDHs. For LDHs with the same cationic laminate, the electrostatic interaction between the intercalated anion and the laminate needs to be greater than that between the laminate and the original intercalated ions if an ion-exchange reaction is to occur. In other words, if the intercalated anion has a bonding interaction with the cationic laminate, the ion-exchange reaction will still occur even if the absorption enthalpy between the two ions is less than that of the original intercalated ions. As shown in Figure 8a, methyl orange was stabilized not only by electrostatic interactions with an LDH layer, but also by π-π stacking between methyl orange molecules, which was considered as the driving force for the substitution of carbonate anions [99].

### 4.2. Layer Spacing

The layer spacing of LDHs plays a crucial role in their corrosion protection performance. It is generally believed that a larger layer spacing facilitates chloride trapping and the release of corrosion inhibitors, endowing the coating with excellent active/passive integrated corrosion protection performance.

Alibakhshi et al. prepared Zn-Al-LDH nanosheets with nitrate (NO_3_^−^) intercalation at various layer spacings by adjusting the pH during the synthesis process, and their structures and layer spacing were characterized (Figure 8c) [20]. The pH value had a great influence on the structure and layer spacing of LDHs during the synthesis process. LDHs synthesized at a pH of approximately 9.5 exhibited a greater layer spacing, and produced a visible protective layer on the surface after immersion of the steel plate for 24 h. Such a protective layer was not observed on mild steel exposed to LDHs synthesized at a pH of 12.5 (Figure 8d). This phenomenon was attributed to the larger layer spacing and more effective ion-exchange behavior, which released more corrosion inhibiting anions, thus generating a protective layer, which directly increased the charge transfer resistance of the LDHs (Figure 8e), achieving excellent corrosion resistance. In addition, the capture of Cl^−^ increased with increasing layer spacing (Figure 8f). The layer spacing is also related to the arrangement of the anions. For example, the layer spacing of LDH-SO_4_^2−^ was larger than that of LDH-CO_3_^2−^, which was attributed to the fact that SO_4_^2−^ was arranged vertically in the LDH laminates, while CO_3_^2−^ was arranged in parallel (Figure 8b) [100].

### 4.3. External Environmental Factors

External environmental factors mainly include pH value and temperature, as well as corrosion-related species. Among these factors, pH plays a key role. The value of pH can affect the layer spacing of LDHs, which directly determines the corrosion protection performance of LDHs. In addition, pH also affects the release rate of anions. Shkirskiy et al. investigated the release kinetics of soluble molybdate inhibitors, and found that solution pH, soluble chlorides, and carbonates affected the release of molybdate ions from intercalated molybdenum in LDHs [101]. They found that molybdate ions were entirely released in alkaline solutions, while the exchange reactions controlled the release of monovalent ions in a neutral environment. In contrast, the release of divalent ions was regulated by surface reactions. This also affected the corrosion protection performance of LDH-reinforced organic coatings. Furthermore, the environmental composition can regulate the corrosion protection performance of LDH-reinforced coatings.

These external environmental factors also influence the driving force, which can indirectly affect the corrosion protection performance of LDHs. The driving force determines the kinetic and thermodynamic feasibility of corrosion protection performance (including chloride trapping and release corrosion inhibitors) [102]. During this process, the driving force is mainly reflected in the concentration gradient or charge difference between the interlayer anions and the corrosion ions presented in the external environment. A higher driving force implies a stronger exchange tendency, which promotes the release of organic or inorganic anions from the interlayers, and also their replacement by corrosion ions, which is crucial for corrosion inhibition performance.

## 5. Computational Simulation

**Figure 9:**
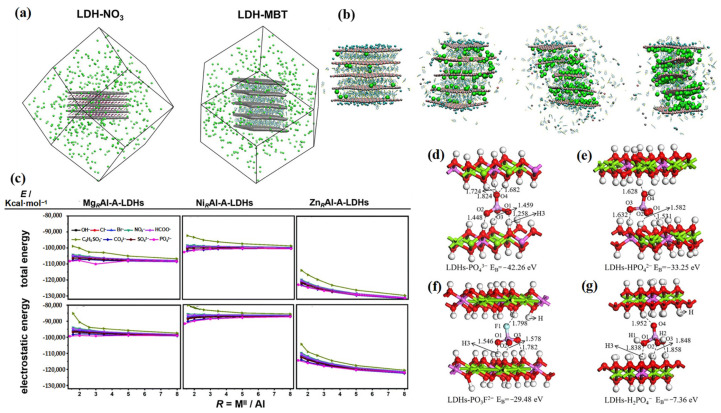
(**a**) Initial models of LDH-NO_3_ (**left**) and LDH-MBT (**right**) were used to perform release kinetics MD simulations. (**b**) Snapshots of different stages of MD simulation for LDH-MBT. Adapted with permission from Reference [103]. (**c**) Total energies and electrostatic energies of MgRAl-A-LDHs, NiRAl-A-LDHs, and ZnRAl-A-LDHs (A = OH^−^, Cl^−^, Br^−^, NO_3_^−^, HCOO^−^, C_6_H_5_SO_3_^−^, CO_3_^2−^, SO_4_^2−^, PO_4_^3−^) as a function of R. Adapted with permission from Reference [102] Copyright 2020, Royal Society of Chemistry. Optimized configurations of (**d**) LDHs-PO_4_^3−^, (**e**) LDHs-H_2_PO_4_^2−^, (**f**) LDHs-PO_3_F_2_^−^, and (**g**) LDHs-H_2_PO_4_^−^. Adapted with permission from Reference [106]. Copyright 2023, Elsevier.

Simulation calculations can help to provide a better understanding of the role and potential mechanisms of LDHs in corrosion protection at the molecular level. For example, molecular dynamics simulations can be used to investigate the initial stages of the ion-exchange process to reveal the corrosion protection mechanism. Leruth et al. developed a classical molecular dynamics model to study the anion-exchange process of LDH intercalated with nitrate and MBT in aqueous sodium chloride solution, to explore the underlying corrosion protection mechanisms (Figure 9a,b) [103]. They found that the concentration of nitrate anions in the middle layer of LDHs decreased by approximately 20%, while the corresponding amount of chloride ions increased within the LDH structure. These phenomena strongly indicated that LDHs were able to trap aggressive molecules from the corrosive environment, while releasing corrosion inhibitors, thus improving the anti-corrosion performance.

Computational simulation provides a better understanding of LDH-based coatings and their underlying protective mechanisms. At present, the most common simulation calculation methods include DFT and molecular dynamics (MD). Zhuang et al. studied the corrosion protection properties and intrinsic mechanisms of LDHs-VB_3_^−^ for rebar through experiments and DFT simulation [104]. Ion exchange testing indicated that LDHs-VB_3_^−^ could effectively capture Cl^−^, thereby protecting the metal substrate. DFT results showed that VB_3_^−^ in LDHs were easily replaced by Cl^−^. The released VB_3_^−^ were tightly absorbed on the metal surface, reducing the attack of Cl^−^, and thus protecting the underlying rebar. Gomes et al. used MD simulation to calculate the interlayer distances of a Mg_2_Al LDH system with Cl-, NO_3_^−^, and CO_3_^2−^ and compared this with the results of XRD [105]. The results indicated strong agreement between the simulation and experimental results, elucidating the mechanisms of corrosion and protection at the molecular level.

To clarify the influence of the charge of the intercalated anions on the chloride trapping efficiency of LDHs, Li et al. simulated the interaction between LDH cationic laminates and anions using DFT, as shown in Figure 9d–g [106]. The binding energies of H_2_PO_4_^−^, HPO_4_^2−^, and PO_4_^3−^ with LDH cationic laminates were calculated to be −7.36 eV, −33.25 eV, and −42.26 eV, respectively. It can be seen that the higher the charge, the stronger the binding ability. Therefore, anions with high charge are more easily exchanged into LDH laminates, facilitating chloride trapping and releasing corrosion inhibitors. This is consistent with the experimental results.

For a better understanding of the impact of the driving force on the corrosion protection performance of LDHs, Zhao et al. used DFT to calculate the total potential energy (binding energy) and electrostatic potential energy in the ion-exchange reaction [102], as shown in Figure 9c. For LDHs containing the same anion, ZnAl-LDH possessed the highest binding energy, which indicated that ZnAl-LDH was more stable than MgAl-LDH and NiAl-LDH. For the same type of LDH, the electrostatic force between the intercalated anion and the laminate cation must be greater than the electrostatic force between the original interlayer ion and the laminate cation, in order for ion-exchange to occur. Therefore, the higher the charge of the interlayer anion, the more negative the total potential and electrostatic energy, making it easier to exchange the anion with a smaller charge. Through computational simulations, we can gain a deep understanding of the corrosion protection functions of LDHs at the molecular level, so as to guide the screening of corrosion inhibitors, facilitating the rational design of LDH-based corrosion inhibitors, and accelerating the development of LDHs with superior anti-corrosion properties.

## 6. Conclusions and Future Perspectives

This review presented an in-depth analysis of recent corrosion protection studies on LDHs, with a particular emphasis on the underlying mechanisms of corrosion protection. LDHs can effectively exert chloride trapping effects and release intercalated corrosion inhibitor ions (self-healing property), forming a protective layer on the metal surface. In addition, LDHs exhibit hydrophobic properties, which are further enhanced by the intercalation of low-surface-energy substances. The primary factors influencing the corrosion protection performance of LDHs were discussed, including the driving force, layer spacing, and external environmental conditions. Furthermore, molecular dynamics simulations and DFT calculations were employed to elucidate the underlying mechanisms at the molecular level. Although LDHs demonstrate significant potential in corrosion protection, several challenges remain, along with opportunities for further improvement.

### 6.1. Synthesis of LDHs for More Effective Response to Various Environments

Based on the synthesis of other 2D nanomaterials, microwave-assisted, ultrasonic-assisted, and electrochemical methods are used to prepare LDH nanosheets. By adjusting various parameters, such as temperature, concentration, and time, the structure of LDHs can be tailored to meet the service requirements of different environments.

### 6.2. Exploring New Insert Materials

New structures and combinations of intercalation materials can be developed through computational materials science and high-throughput experimental methods, such as first-principles calculations, molecular dynamics simulations, and DFT. For example, anions with higher charge density and stronger binding ability may help to further enhance the anti-corrosion properties of LDHs.

### 6.3. Exploring Multifunctional Integrated LDH-Reinforced Composite Coatings

The development of high-performance, multifunctional integrated coating systems is a critical direction in future research on LDH-reinforced organic coatings. By designing new coatings with excellent anti-corrosion, anti-biofouling, intelligent self-healing, and early corrosion monitoring functions, they are expected to achieve long-term corrosion protection in challenging environments.

### 6.4. Development of Advanced Multifunctional Integrated Coatings Represents Urgent Requirement for Advancing LDH-Reinforced Coating Engineering

Plant extracts, such as tea polyphenols, can be used as raw materials for the preparation of LDHs. Additionally, plant-derived corrosion inhibitors can also be developed to create functionalized LDH carriers. Specifically, the chelation effect of plant polyphenols can be utilized to modify LDH-based coatings, achieving chloride ion capture and intelligent release of corrosion inhibitors. By developing hybrid materials that integrate green corrosion inhibitors with LDHs, and improving the adhesion of coatings through both physical and chemical bonding, long-term corrosion protection can be achieved.

### 6.5. Machine Learning-Assisted Preparation of High-Performance LDH-Reinforced Composite Coatings

Machine learning can be utilized to regulate various parameters, such as thickness and concentration, enabling precise control over complex structural surfaces, while simultaneously reducing costs and energy consumption. Additionally, it can expedite the optimization of LDH formulations and processes, facilitating the machine learning-assisted preparation of high-performance LDH-reinforced composite coatings.

## Figures and Tables

**Table 1:** Anti-corrosion performance of LDHs with different intercalating ions.

LDH Type	Interlayer Anion	Morphometric	Solution	Corrosion Current Density A/cm^2^	Immersion Time	|Z|_0.01 Hz_ Ω cm^2^	Refs.
ZnAl-LDH	NO_3_^−^	Film	3.5 wt% NaCl	10^−8^	14 days	1.6 × 10^7^	[82]
ZnAl-LDH /EP	VO_3_^−^	Powder	0.05 M NaCl	N/A	4 months	>10^6^	[69]
ZnAl-LDH /EP	C_6_H_5_COO^−^	Powder	3.5 wt% NaCl	4.9 × 10^−6^	24 h	>10^5^	[83]
ZnAl-LDH /EP	PO_4_^3−^	Powder	3.5 wt% NaCl	4.23 × 10^−9^	100 days	≈10^7^	[73]
ZnAl-LDH /EP	MoO_4_^2−^	Powder	3.5 wt% NaCl	2.94 × 10^−9^	100 days	≈10^7^	[73]
ZnAl-LDH /sol–gel	V_2_O_7_^4−^	Powder	3.5 wt% NaCl	1.71 × 10^−6^	14 days	>10^6^	[70]
ZnAl-LDH /EP	Methionine	Powder	3.5 wt% NaCl	9.98 × 10^−6^	85 days	>10^8^	[84]
ZnAl-LDH	VOx and La	Film	3.5 wt% NaCl	5.19 × 10^−9^	24 h	>10^6^	[78]
ZnAl-LDH	SDS	Film	3.5 wt% NaCl	2.01 × 10^−8^	12 h	7 × 10^4^	[85]
ZnAl-LDH /EP	DEDTC	Film	3.5 wt% NaCl	N/A	24 h	≈10^6^	[86]
ZnAl-LDH	MBT	Powder	0.05 M NaCl	N/A	7 days	>10^5^	[49]
ZnAl-LDH /ZRE	MBT	Powder	3.5 wt% NaCl	N/A	25 days	≈10^4^	[74]
ZnAl-LDH /PEO	Fumarate	Film	3.5 wt% NaCl	1.5 × 10^7^	10 days	≈10^5^	[87]
MgAl-LDH	MBT	Powder	0.05 M NaCl	N/A	7 days	>10^4^	[49]
MgAl-LDH /EP	F^−^	Powder	3.5 wt% NaCl	N/A	30 days	>10^8^	[80]
MgAl-LDH	WO_4_^2−^	Film	3.5 wt% NaCl	7.44 × 10^−6^	7 days	>10^4^	[88]
MgAl-LDH	CO_3_^2−^	Film	5 wt% NaCl	4.82 × 10^−11^	10 days	N/A	[89]
MgAl-LDH /EP	CO_3_^2−^	Powder	3.5 wt% NaCl	6.52 × 10^−8^	48 h	>10^5^	[90]
MgAl-LDH	NO_3_^−^	Film	3.5 wt% NaCl	3.626 × 10^−7^	21 days	4.77 × 10^7^	[91]
MgAl-LDH /EPSP	PO_4_^3−^	Powder	3.5 wt% NaCl	4.8 × 10^−6^	24 h	≈10^4^ (artificial defect)	[92]
MgAl-LDH /EP	NO_2_^−^	Powder	3.5 wt% NaCl	N/A	25 days	3.52 × 10^7^	[29]
MgAl-LDH	Sodium laurate	Film	3.5 wt% NaCl	4.33 × 10^−9^	7 days	≈10^7^	[93]
MgAl-LDH	Oxalate	Film	3.5 wt% NaCl	N/A	48 h	>10^4^	[94]
MgAl-LDH	PPA	Film	3.5 wt% NaCl	2.47 × 10^−9^	36 h	≈10^6^	[95]
MgAl-LDH /EP	Methionine	Powder	3.5 wt% NaCl	5.8 × 10^−6^	60 h	≈10^4^	[84]
MgAl-LDH /EP	MoO_4_^2−^ and BTA	Powder	3.5 wt% NaCl	N/A	120 days	>10^7^	[6]
LiAl-LDH	Asp	Film	3.5 wt% NaCl	10^−8^	20 days	4.41 × 10^5^	[79]
LiAl-LDH	L-aspartic	Film	3.5 wt% NaCl	3 × 10^−8^	5 days	3.6 × 10^6^	[66]
MgFe-LDH	8HQ	Film	3.5 wt% NaCl	N/A	7 days	>1.5 × 10^4^	[64]
NiAl-LDH /PU	PO_4_^3−^	Powder	3.5 wt% NaCl	N/A	14 days	≈10^11^	[71]
NiAl-LDH	VO_x_	Powder	3.5 wt% NaCl	1.13 × 10^−6^	24 h	2.54 × 10^5^	[96]
CaAl-LDH	MnO_4_^−^	Film	3.5 wt% NaCl	N/A	28 days	≈10^5^	[97]

## Data Availability

No data were used for the research described in the article.
